# Supplementary material for: Are carbon emissions trading and green financial instruments synergistic? -Comprehensive quantitative research based on content analysis
Source: PLoS One. 2024 Mar 7;19(3):e0298601. doi: 10.1371/journal.pone.0298601 (PMC10919601; doi:10.1371/journal.pone.0298601)
Supplement: S1 Data — (ZIP) [file pone.0298601.s001.zip › Data/地级市Z_F工作报告.zip!/╔┬╬≈/╧╠╤⌠╩╨/└┤╫╘╣1⁄2╓┌║┼í╛┬φ┐╦╩2╛▌═°í┐_2019.docx]

各位代表：­­­­

现在，我代表市人民政府向大会作工作报告，请予审议，并请市政协委员和列席人员提出意见。

一、2018年工作回顾

过去一年，在省委、省政府和市委的坚强领导下，我们坚持以习近平新时代中国特色社会主义思想为指导，紧扣追赶超越和“五个扎实”要求，统筹稳增长、促改革、调结构、惠民生、防风险，实施“一补三稳五强化”工程，抓项目，稳工业，促县域，全市经济平稳增长、质量提升，生产总值完成2376.5亿元、增长7%；地方一般公共预算收入完成89.1亿元、增长15%，税收占比提高到81.4%；城乡居民人均可支配收入分别达到33364元、10893元，增长8.1%、9.1%。主要做了以下工作：

一是突出工业抓转型，结构调整迈出新步伐。8.6代液晶面板项目实现满产，聚集配套企业22户，冠捷电视机搭乘“长安号”销往“一带一路”沿线国家。雷丁秦星5万辆商用车项目建成投产、25万辆乘用车项目加快建设，咸阳成为全省汽车产业 “一体两翼”布局重要板块。扎实推进工业发展“3+1111”工程，100个重点工业项目完成投资234.8亿元，新增规上工业企业51户。规模以上工业增加值增长7.2%。农业供给侧结构性改革取得新进展，全年粮食、水果、蔬菜、肉蛋奶产量分别达到160.2万吨、418.36万吨、309.9万吨、55.79万吨，旱腰带葡萄和石榴分别达到19.5万亩、3.3万亩，奶山羊存栏量占全省1/3、全国1/5，“咸阳马栏红”苹果、“武功小子”猕猴桃、泾阳茯茶深受消费者认可，农民专业合作社、家庭农场、农业产业化龙头企业分别达到6248个、1535个、212个。多措并举减轻春季低温霜冻灾害损失，严防严控非洲猪瘟疫情发生。第三次全国农业普查荣获全国先进集体。实施商贸服务业、电子商务提升行动计划，长武、永寿成功创建国家级电子商务进农村综合示范县。大力发展全域旅游，兴平、彬州、礼泉、泾阳荣获“全国百佳乡村旅游目的地”称号，彬州拜家河村入选“中国最美乡村”，全市旅游综合收入385.96亿元、增长32.09%。举办购物美食节扩大消费，社会消费品零售总额增长11%。

二是扭住项目抓投资，动能转换实现新突破。出台扩大有效投资考核奖惩办法，压实工业项目招商引资任务，全社会固定资产投资完成1968.01亿元、增长9%，民间投资增长20.5%。155个市级重点项目完成投资552.4亿元、占年计划的114.65%，49个项目已经竣工，法士特、陕煤乙二醇等重大项目加快建设，国能CFB低热值示范发电项目获批。西银高铁加快建设，银百高速旬邑至陕甘界全面开工，7条国省干线公路升等改造工程进展顺利，新建改建县乡公路333.4公里，西咸综合客运枢纽建成投用，交通物流优势进一步强化。东庄水库、泾河综合治理等重大水利工程开工建设，水利设施支撑发展能力进一步增强。招商引资到位资金707.1亿元，实际利用外资9016万美元。强化创新驱动，建立国家级院士专家工作站5个，新增高新技术企业32家，高技术产业工业增加值增长14.5%。

三是精准施策抓脱贫，国定贫困县有望全部摘帽。全面推行“六四四”工作法，全年投入扶贫专项资金10.19亿元，易地移民搬迁全省率先完成、搬迁户全部入住，农村危房改造竣工5332户，“油返砂”道路整治959.9公里，建成标准化卫生室84个、农村安全饮水项目312处，精准扶贫大数据平台被评为“2018数字中国示范案例”。中行定点扶贫、咸阳·泰州协作扶贫力度持续加大，“北四县”4家中银富登村镇银行开业。正大养殖项目加快建设，电商、光伏、食用菌种植等扶贫产业加快发展，贫困户中长期产业覆盖率达到65%，贫困村集体经济实现全覆盖。全年脱贫7.39万人，退出贫困村478个，4个国定贫困县退出已达标。

四是强化措施抓治霾，环境保护取得新进展。推行“五项清单”夯实责任，开展周日专项督查传导压力，推行网格化管理打通落实“最后一公里”。重拳整治“煤尘车”污染，分类推进“散乱污”企业治理，超额完成规上企业煤炭削减任务；成功争取全国冬季清洁取暖试点，中央财政连续三年每年支持3亿元，铁腕治霾向纵深推进，全年空气质量综合指数下降9.35%，PM2.5平均浓度下降12.66%。举一反三抓好中央和省委环保督察反馈问题整改，北部矿山生态修复治理成果持续巩固，4个国考监测断面水质稳定达标。开展农村人居环境整治三年行动，89.9%的行政村生活垃圾、41%的行政村生活污水得到有效治理，无害化卫生厕所普及率达到44%。“大棚房”整治取得阶段性成果。国土整治新增耕地2.5万亩。在全省率先实行“林长制”，创建森林城市扎实开展。

五是规划引领抓城建，城乡融合发展呈现新局面。认真落实省政府批复的咸阳城市总体规划，大西安（咸阳）文体功能区发展框架基本形成，奥体中心、如意塔、双照湖深受群众喜爱，物流、会展等产业加速聚集；渭河南槽治理工程推进顺利；暖温带森林博览园开工建设，“三城两带”城市空间格局基本形成。156个城建项目进展顺利，完成秦皇路等10条道路街景改造和6条道路绿化，彩虹北路北延段等9条城市道路建成通车，城市雨污分流率比上年提高6.7个百分点。城市精细化管理水平进一步提升，全国文明城市、国家园林城市、国家卫生城市通过复审。乡村振兴开局良好，“十大行动”“百优示范项目”启动实施。加强镇政府服务能力建设，分片推进县域发展，彬州成功撤县设市，三原进入全省“十强县”，全省“十强县”咸阳占3席。

六是优化环境抓营商，改革开放激发新活力。持续深化“放管服”改革，自主取消下放调整市级行政审批事项21项，新增“最多跑一次”事项235项，9个县市区实现“一枚印章管审批”，市级部门行政审批事项实现一个科室办理全覆盖，政务服务“一网通办”上线运行。商事制度改革突出宽进严管，“双随机、一公开”监管模式加快推行。开展“大走访”活动，多措并举优化营商环境。全年新增各类市场主体40871户、增长22.4%，非公经济占比达到53.5%。扎实推进农村土地“三权分置”改革试点和农村集体产权制度改革，11个县市区农村产权交易中心建成投用。实施扩大开放行动计划，首届进口博览会签约额全省第二，进出口总额73.25亿元，增长69.4%。

七是聚焦民生抓实事，群众生活有了新改善。民生支出317.2亿元，占财政支出的83.5%。30件惠民便民实事完成投资137.6亿元。提前一年实现基础教育“双高双普”目标，英才学校建成招生，新渭城中学、彩虹第二中学等城区学校工程加快建设。新增义务教育阶段学位1.17万个，普惠性幼儿园占比提高到81.07%。创建省级文化产业示范基地4个。电影《信·守》获得3项大奖。完成市县两级第二轮地方志编纂任务。成功举办省十六运，公共体育设施明显改善。启动“健康咸阳”建设，深化综合医改，我市被评为全省现代医院管理制度改革先进市。新农合参合率99.1%，统筹区域内政策性报销比例82.42%，超出省考指标9.42个百分点。将17类抗癌药品纳入医保，城镇居民医保政府补助提高到500元。企业职工和城乡居民养老金标准分别提高151元、23元。城镇新增就业5.44万人，城镇登记失业率3.06%。居家和社区养老服务改革试点扎实推进，城乡低保做到应保尽保。棚户区改造项目开工13537套，建成8880套、分配公租房5620套。“扫黑除恶”专项斗争战果丰硕，成功破获“1·16”系列盗掘古塔古墓葬等大案要案，平安建设扎实推进。扎实开展安全生产“三项攻坚”行动，未发生较大以上安全生产事故，社会大局保持和谐稳定。

双拥、残疾人、红十字、妇女儿童等事业取得了新进步，民族、宗教、外事、侨务、对台、审计、地震、人防、气象、档案、盐务等工作取得了新的成绩。

各位代表，这些成绩的取得，是省委、省政府和市委坚强领导的结果，得益于市人大、市政协的监督和支持，是广大干部群众共同奋斗的结果。在此，我代表市人民政府，向全市广大干部群众，向各民主党派、人民团体，向驻咸人民解放军、武警官兵和政法干警，向关心支持咸阳发展的各界人士，表示衷心的感谢和崇高的敬意!

在肯定成绩的同时，我们也清醒认识到，全市经济社会发展中仍然存在不少矛盾和问题。主要是，工业经济结构偏重化工，大项目、好项目后劲不足，县域经济支撑不强。空气质量指标虽有所改善，但秋冬季雾霾频发，铁腕治理任务艰巨。一些民生问题还没有解决到位，解决教育“城市挤”“农村弱”以及“看病贵”“出行堵”“停车难”问题需要进一步加大力度。营商环境的竞争力还不够强。对此，我们将对症下药，综合施策，在化解问题中提升群众幸福感、提升发展质量。

二、2019年目标任务

2019年是新中国成立70周年，是全面建成小康社会关键之年。做好今年政府工作，意义重大、使命光荣。今年全市经济社会发展的总体要求是：以习近平新时代中国特色社会主义思想为指导，坚持稳中求进工作总基调，坚持新发展理念，坚持以供给侧结构性改革为主线，全面落实“五个扎实”要求和“五新”战略任务，深化市场化改革、扩大高水平开放，持续打好攻坚战，大力实施项目带动、结构升级、生态优先、民生增惠、党建领航五大方略，围绕“六稳”统筹推进稳增长、促改革、调结构、惠民生、防风险工作，打造全省“三个经济”先行区，决胜全面建成小康社会关键年，奋力实现新时代追赶超越新作为，以优异成绩庆祝中华人民共和国成立70周年。

主要预期目标是：生产总值增长6.5%—7%，规模以上工业增加值增长7%左右，全社会固定资产投资增长9%左右，社会消费品零售总额增长10%左右，地方一般公共预算收入同口径增长7%左右，城镇居民人均可支配收入增长7.5%左右，农村居民人均可支配收入增长8%左右。

实现上述目标，困难不少、挑战巨大。宏观经济稳中有变、变中有忧，经济下行压力有所加大。从长远看，我国发展仍处于并将长期处于重要战略机遇期，随着“一带一路”、关中平原城市群及全省发展“三个经济”等重大政策及战略的深入实施，咸阳各种优势叠加放大；尤其是，经过持之以恒调整产业结构、优化城市空间结构，全市经济新动能成长在提速，发展重心转换在加快。分析机遇与挑战，我们优势凸显，潜力正在转换为动力，全市上下要坚定信心，通过担当实干放大机遇，积蓄追赶超越的强大力量。在工作中，重点把握以下几点：

一是坚持稳中求进工作总基调。认真落实“六稳”要求，确保经济运行在合理区间。坚持“巩固、增强、提升、畅通”八字方针，扭住深化供给侧结构性改革这条主线，在增强微观主体活力上采取更有力措施，在调整经济结构上迈出更大步伐，稳中求进，以进固稳，推进高质量发展不断取得新成效。

二是紧盯全面建成小康社会历史任务。对标全面建成小康社会，缺什么就补什么，哪里弱就加强哪里，着力提高城乡群众收入水平，不断提高公共服务质量，为全面建成小康社会收官打下决定性基础。

三是全力推行经济工作项目化。以项目检验干部、以项目体现担当。用足用活国家加大基础设施领域补短板力度政策机遇，进一步强化招商引资工作，大抓项目，抓大项目，以项目扩投资、调结构、补短板、增后劲，厚植发展优势。

四是提高驾驭市场经济的能力。运用市场化法治化手段，激活市场活力。持续优化营商环境，增强对生产要素的聚集能力。按照省政府批复的城市总体规划，立足关中平原城市群核心区，加快渭河汽车工业走廊、临空经济产业带“一廊一带”建设，打造“三个经济”先行区。

五是着力防范化解重大风险。坚持底线思维，增强忧患意识，提高风险化解能力，完善决策风险评估、研判、防控等机制，下好“先手棋”，打好“主动仗”，着力防范化解各类重大风险，保持经济持续健康发展和社会大局稳定。

三、2019年重点工作

（一）全力以赴保增长，力促经济高质量发展

全力稳工业。继续实行市县领导包抓重点企业制度，强化工业政策供给，工业发展专项资金重点向培育产业集群、推进技术创新、园区发展倾斜。着力在“优”字上下功夫，确定50户行业龙头企业，精准帮助解决人才、融资、用地等方面问题，支持企业拓展市场、扩大产能、带动集群，以优势企业稳工业。着力在“转”字上求突破，运用市场化手段，因企施策，服务脱困一批、技改转型一批、兼并重组一批、引资接盘一批，推进停产半停产企业重新焕发活力。着力在“增”字上见成效，抓好总投资394.2亿元的100个重点工业项目建设，促进法士特项目一期投产、二期开工，促进陕煤乙二醇项目年内试车。继续实施“个转企、小升规”培育计划，全年新增规模以上工业企业70户。

全力扩投资。继续强化对项目工作考核，建立项目谋划、建设、服务联动推进机制。今年安排市级重点项目334个、总投资2253亿元、年度投资809亿元。对这些项目，我们将严格包抓责任，加强项目管理，优化服务保障，促进西北特种电缆等102个续建项目按期建成，促进浩泽环保智慧产业园等232个新建项目全部开工。用足用活国家基础设施领域补短板政策，积极扩大政府债券规模；创新PPP融资模式，吸引民间投资，持续加大基础设施、社会民生、乡村振兴、生态环保等领域投资。

全力促消费。出台城市商业网点规划，鼓励发展便利店、社区菜店等社区商业，加大农贸市场改造力度，提升特色商业街区和步行街，培育发展城市商圈。加强对养老服务的政策扶持，引导市场深耕“银发经济”。依托丰富的中医保健资源和医疗卫生资源，支持医养康养融合发展，扩大健康消费。发挥市民文化中心、奥体中心作用，市场化运作文化展演、体育联赛，培育文化体育消费。多渠道增加优质供给，促进教育培训托幼消费。健全农村流通网络体系，促进农村消费梯次升级。加强充电桩建设，促进新能源汽车消费。开展智慧咸阳再出发，带动信息消费。支持星级酒店建设，打造一批特色化品牌化节会展会，培育新的消费热点。加强市场监管，开展优质诚信示范店创建，让群众安心消费、放心消费。

（二）久久为功促转型，打造“三个经济”发展先行区

随着西安获批国家中心城市，咸阳具有的区位优势、一流的综合交通枢纽优势、积淀深厚的文化优势、门类较全的产业优势将更加凸显。要抢抓省上建设临空经济示范区机遇，依托国际机场，与空港新城协同发展，以大西安（咸阳）文体功能区为核心，启动临空经济产业带建设，带动制造业高端化、农业品牌化、服务业特色化，打造全省“三个经济”发展先行区。

做大做强支柱产业。围绕打造电子信息千亿产业集群，以高新区为主战场，促进8.6代液晶面板22户配套企业达产达效，力争3年内再招引100家电子信息企业。围绕打造新能源汽车千亿产业集群，推进雷丁秦星25万辆乘用车项目、武功沃德10万辆新能源乘用车项目建成投产，继续加大汽车龙头企业引进力度；开工建设百万平米智能制造厂房，吸引汽车零部件配套产业聚集发展，打造渭河汽车工业走廊。围绕打造高端装备制造500亿产业集群，推进装备制造园体制机制创新、基础设施建设提速，加快正泰智能电气系列项目建设，着力在引进龙头企业、培育产业集群上取得重大突破。围绕打造生物医药500亿产业集群，依托高新区医药产业园和旬邑中医药健康产业园，支持步长、康惠、海天、摩美得等企业做大做强，加快中港万海细胞制备中心等项目建设，提升医药产业竞争力。围绕推进煤炭“三个转化”，加快亭口水库输配水工程建设，开工建设彬州通用机场，提升彬州新民高端能源化工园承载能力，加快长武60万吨甲醇技改项目建设，促进国能CFB低热值示范发电项目尽快开工，不断蓄积彬长旬高端能化基地发展势能。培育高端橡胶产业集群，推进建材产业绿色转型，发展壮大新材料产业。扎实开展“三品”专项行动，提升食品、纺织等产业竞争力。培育发展机器人、智能制造等新兴产业，优化工业经济结构。

发展壮大现代服务业。依托区位交通优势，优化物流园区布局，加大物流龙头企业引进，完善物流网络体系，做强现代物流。扎实开展全国网络市场监管与服务示范区创建，推进电子商务龙头聚集、业态创新，鼓励发展农村电商，积极发展跨境电商，打造全国生鲜电商聚集地。加强全域旅游示范市创建，创新模式推进“旅游+”，支持乾陵创建5A级景区，促进彬州大佛寺文化景区加快建设，布局建设自驾营地，打造一批旅游精品线路和大景区。培育壮大文化产业市场主体，带动文化资源创造性转化、文化产业创新性发展。支持大数据以及融资租赁、检验检测、人力资源服务等服务业发展壮大，助推产业转型升级。

扭住招商引资不放松。强化质量效益导向，突出工业项目、高科技项目，加大考核奖惩力度，压实招商引资责任。继续加大对东南沿海地区招商力度，积极拓展日韩、新加坡及香港地区招商。发挥商（协）会及专业机构作用，扎实开展以商招商、平台招商、专题招商以及产业链招商。建立项目跟踪和精准服务机制，确保招商引资实现到位资金800亿元、引进外资1亿美元。积极扩大对外开放，加强中韩、中哈产业园建设，发展外向型经济。

坚持推进科技创新。立足科技成果转化，以“科技创新六大工程”为抓手，深化与高校及科研院所合作，加大工程技术中心、重点实验室、院士工作站等创新平台和工业互联网平台引进支持力度，扶持高新区筑梦·创享空间等孵化平台发展，实施好“双导师制”，促进创新链、资本链、产业链对接融合。扎实推进质量强市战略，加大瞪羚企业、科技小巨人企业、高新技术企业培育力度，支持大中小企业协同创新、融通发展。出台军民融合发展政策措施，培育“民参军”“军转民”示范企业，支持创建军民融合示范基地。发挥人才基金“四两拨千斤”作用，不断增强创新驱动力。

（三）打好三大攻坚战，奋力补齐发展短板

坚决打好防范化解重大风险攻坚战。未雨绸缪，综合施策，有效防范管理处置各种风险。实行政府举债融资负面清单制管理，建立健全政府举债终身问责制度和债务问题倒查机制，坚决遏制债务增量，稳妥化解债务存量。推进融资平台公司市场化转型，从严规范融资平台管理，坚决查处各类违法违规举债担保行为。严厉打击非法集资、金融诈骗、恶意逃废债等违法行为，加强互联网金融风险专项排查整治。

坚决打好精准脱贫攻坚战。围绕高质量完成年度2.5万人脱贫、72个贫困村退出任务，工作力量、扶持重心向旱腰带地区倾斜，向深度贫困村和特殊困难群体帮扶汇聚。对已经退出的贫困村和贫困户，做到脱贫不脱钩、脱贫不脱政策、脱贫不脱帮扶；对收入水平略高于建档立卡贫困户的群众，精准帮助解决难题。扎实推进“八个一批”政策落实，更加重视发展壮大稳定脱贫产业，扶持旱腰带地区葡萄和石榴产业发展，推进正大养殖以及“安居+乐业”社区工厂等扶贫项目发挥效益，确保贫困群众有稳定收入来源。深化“三变”改革，创新利益联结机制，让贫困群众分享产业链收益。加强交通、水利、电力等基础设施建设，增强发展能力。以项目为抓手，深化中行定点扶贫、咸阳·泰州协作扶贫以及“3+X”帮扶。开展形式多样的扶志扶智活动，增强贫困群众内生动力。

坚决打好污染防治攻坚战。以打赢蓝天保卫战为重点，聚焦“煤尘车”和“散乱污”企业，继续实行“五项清单”制度，全面运行网格化管理，不断提升精细化治理水平；扎实推进冬季清洁取暖试点工作，足额兑现补助政策，整镇推进，让空气更清新，让群众得实惠；按照“关停淘汰一批、改造升级一批、搬迁入园一批”原则，因企施策治理“散乱污”企业；实施长庆石化、兴化等4户化工企业提标改造，加大力度调整产业结构、能源结构、运输结构，早日迎来空气质量稳定好转的拐点。坚决打好青山、碧水、净土保卫战，支持光伏领跑者计划实施，协同推进北部矿山生态修复治理与产业发展；细化落实河长制、湖长制，加快泾河干流综合治理进度，加强城镇污水处理设施建设，消灭黑臭水体，促进河流水质稳定向好；推进废弃物资源化利用，加强农业面源污染防治。扎实开展国家森林城市创建，完成营造林30万亩，绿化道路300公里。加快建立“三线一单”环境管控体系，严格执行生态环境损害赔偿制度，从制度上根源上遏制污染、改善生态。

（四）加快新型城镇化步伐，构建现代城镇体系

提升中心城市品质。加快完善城市规划体系，强化规划引领约束作用。加快大西安（咸阳）文体功能区发展，启动地下人防工程以及市特教学校、陕工职院新校区等公共服务项目，加快锦秀山公园、西照湖等基础设施项目建设，着力发展临空经济、会展经济、文化创意产业，提升聚集带动能力。启动咸阳湖景区改造提升和渭河南岸河堤加固改造工程，全面建成渭河水生态带。启动市植物园建设，推进汉帝陵森林景观带建设。开工高科一路渭河大桥，打通西宝高速沣河ETC出口，促进西咸便捷高效联通。继续实施道路对接、疏堵保畅、品质提升、立体绿化、精细管理“五大工程”，抓好年度投资82.5亿元的127个城建项目。创建公交都市，坚持公交优先，市县两级再投放新能源公交车600辆。开工建设东风路综合改造、珠泉路道路排水工程，新建支路5条，打通“断头路”10条，建设立体停车场（库）10个、人行天桥4座，持续加大“出行堵”“停车难”问题解决力度。建设绿地广场12个，加强雨污分流改造，提升绿化亮化美化水平，让城市更宜居、更现代。扩大文明市场、文明社区覆盖面，持续深化城乡联动，不断巩固提升全国文明城市创建成果，让文明新风在咸阳蔚然成风。

借力关中平原城市群加快发展。认真落实省政府关中平原城市群发展规划实施方案，坚定不移推进西咸一体化，开工建设地铁1号线三期；服务保障阎良至机场、乾县至法门寺2条城际铁路加快建设；积极争取利用陇海线开通西安咸阳城际快车。加快融入区域发展大格局，服务保障西银高铁以及银百高速旬邑至陕甘界、菏宝高速旬邑至凤翔段建设，启动福银高速、连霍高速连接线工程，建成西宝高速兴平阜寨出口工程，抓好10条国省干线升等改造。在市域北部谋划建设通用机场、货运机场、运煤专线、矿区路网连接项目，在南部加密道路连接路网，谋划建设轨道交通项目，以交通设施大联通为发展“三个经济”、融入“一带一路”奠定基础。

做大做强县域经济。坚持“两手抓”和“园区化”，一手抓资本密集型项目强县，一手抓就业密集型项目富民，加强对县域招商引资、工业发展、园区建设的考核，加快县域工业园区建设，全面学习蔡家坡经验，加强标准化厂房建设，提升产业园区承载力，推进县域争先进位。支持秦都、兴平、武功联手打造渭河汽车工业走廊，支持礼泉与大西安（咸阳）文体功能区联动发展，支持泾阳优先发展临空经济，梯次推进三原、乾县撤县设市，支持渭城做优做强城市经济，支持永寿、淳化发展特色工业，支持彬长旬煤炭转化、多元发展。

（五）实施乡村振兴战略，巩固全省农业龙头地位

坚持农业农村优先发展，加大力度实施乡村振兴战略，扎实推进“十大行动”和“百优示范项目”，不断迈出农业农村现代化新步伐。

加快发展优质高效、生态观光现代农业。实施藏粮于地、藏粮于技战略，保障区域粮食安全。持续深化农业供给侧结构性改革，发挥“咸阳马栏红”品牌的引领作用，促进苹果种植结构优化、效益提升。鼓励发展设施蔬菜和高山冷凉蔬菜，全产业链推进奶畜产业，支持发展猕猴桃、食用菌、中药材、苗木花卉等特色产业。承办第八届北京茶博会，提升“咸阳茯茶”知名度、竞争力。启动食用农产品合格证制度试行工作，扩大优质绿色农产品供给。引进培育龙头企业、农民专业合作社、家庭农场等新型农业经营主体，带动农业与电商、旅游等产业融合发展。支持农业保险扩面增品提标。培育植保无人机产业链，示范推进苹果生产全程机械化，提升农业机械化示范区建设水平。毫不放松地抓好非洲猪瘟防控工作。坚决清理整治“大棚房”。推进引汉济渭工程建设，实施东庄水库施工供水工程国土综合整治项目，加强农田水利基础设施建设。

建设美丽宜居乡村。学习浙江“千村示范、万村整治”先进经验，压实县级主体责任，重点抓好垃圾污水治理、厕所革命和村容村貌提升，持续改善农村人居环境。推进“四好农村路”建设，改造建设县乡公路230公里、通村公路“油返砂”整治500公里。扎实推进饮水安全、农网改造、气化农村等工程，推动农村基础设施提档升级。发挥示范村引领，推进“多规合一”，加强村庄设计，引导村庄体现关中风情、彰显特色风貌。建立健全自治、法治、德治相结合的乡村治理体系，培育文明乡风、淳朴民风，遏制大操大办、厚葬薄养、人情攀比等陈规陋习，让农村美在环境、美在人文。

创新农业农村体制机制。深化农村土地制度改革，探索“三权分置”多种实现形式，引导小农户对接大市场。全面开展农村集体经济组织成员资格认定，不断深化农村集体产权制度改革。完善城乡融合发展体制机制，鼓励引导工商资本参与乡村振兴，切实保障农民合法权益，多渠道增加农民收入。

（六）持续深化改革，不断增强发展活力

扎实推进供给侧结构性改革。巩固“三去一降一补”成果，严防“地条钢”死灰复燃，运用市场化、法治化手段推动“僵尸企业”出清。进一步降低营商成本，落实更大规模减税降费政策，全面实施修改后的个人所得税法，适当降低社保费率。积极争取扩大直供电交易规模，力争全年达到15亿度以上；推行工业用地弹性出让；有效降低物流成本，让企业有明显获得感。

持续深化“放管服”改革。深化行政审批制度改革，再梳理取消下放一批“含金量”高的行政审批事项，县市区全面实行“一枚印章管审批”，扩大“最多跑一次”事项覆盖面。优化“互联网+政务服务”，上线运行政务服务综合APP，市县级政务服务事项网上可办率不低于70%。优化提升实体政务大厅综合窗口服务功能，做到服务事项“应进全进”。持续深化商事制度改革，加快实现“双随机、一公开”全覆盖、常态化，严格全程监管食品、药品和特种设备等重要领域，包容审慎监管新兴产业。优化生态环境、安全生产、市场监管执法检查方式，提升执法监管法治化、程序化、规范化水平。

持续激发市场主体活力。深化国资国企改革，全面摸清国有资产底数，推进国有资产统一管理、分类监管。支持国有资本投资运营公司做专、做优、做强，确保国有资本保值增值。推进“三供一业”分离移交，剥离国有企业办社会职能。毫不动摇支持非公有制经济发展，确保“非禁即入”落到实处。切实保护民营企业合法权益。成立民营企业维权投诉中心，开展清欠、减负、化解产权纠纷等行动。提高金融服务实体经济的能力，完善融资担保体系，加强政银企对接。设立民营企业纾困基金，健全降低中小微企业融资成本的奖补机制。鼓励企业上市，发挥股权市场作用，扩大直接融资。以争创“零投诉”营商环境为导向，实施优化提升营商环境工作三年行动计划，建立投诉问题办理、回复、督查机制，加大正向激励和反向惩戒力度。推行企业24小时平静工作日制度。构建亲清新型政商关系，营造亲商安商富商环境。

（七）坚持以人民为中心，不断增进民生福祉

做好就业和社会保障工作。把稳就业放在突出位置，以创业促就业，重点做好高校毕业生、退役军人、就业困难人员、贫困劳动力、返乡农民工等群体就业工作。落实全民参保计划，深化基本养老保险制度改革；完善城乡居民基本医疗保险和大病保险政策，扩大异地就医即时结算范围，把更多救命救急的好药纳入医保，严厉打击套保骗保行为；调整提高城镇退休人员基本养老金水平，建立城乡居民养老保险待遇确定和基础养老金正常调整机制。认真做好退伍军人事务，巩固军政军民团结。稳步提高低保、优抚救助水平，做好留守儿童、困境儿童、留守妇女、空巢老人和残疾人关爱工作。完善工资收入分配制度，健全职工工资正常增长机制。开展“治欠保支”行动，严厉打击拖欠农民工工资违法行为。持续加大棚改力度，完善棚改配套基础设施。

提升公共服务质量。认真贯彻全国全省教育大会精神，坚持立德树人根本任务，落实“三个优先”要求。建成彩虹第二中学，加快新渭城中学、高新学校、梦桃学校建设，普惠性幼儿园占比提高到85%以上。实施“名校+”战略，促进教育优质均衡发展。开工建设咸阳职院二期工程，建设职教强市。服务在咸高校发展，加强校地合作。深化教育体制机制改革，完善教育评价体系，加强师德师能建设，努力办人民满意的教育。扎实推进“健康咸阳”建设，持续扩大“健康细胞”覆盖面。持续深化综合医改，深入推进分级诊疗。实施改善医疗服务行动计划，加快市中心医院医技住院综合楼等项目建设，着力解决“看病难”“看病贵”问题。加强疫苗全流程监管，确保疫苗接种安全。优化妇幼健康全周期服务，扩大农村适龄妇女免费“两癌”筛查项目覆盖面。开展地方病防治专项行动，确保到2020年基本消除大骨节等地方病危害。加大体育惠民工程实施力度，加快市体育场改造工程，推进全民运动健身模范市创建。加强文物保护利用和文化遗产保护传承。深化文化体制改革，实施文化惠民工程，举办第二届咸阳文化艺术节。

加强社会治理。不断掀起“扫黑除恶”专项行动凌厉攻势，实施“雪亮工程”，依法惩治犯罪活动。推进城乡社区治理和服务创新，畅通群众诉求通道，从源头上防范化解矛盾纠纷。推进国家食品安全示范城市创建，扎实开展农村假冒伪劣食品专项整治行动，保障群众“舌尖上的安全”。加大保健品市场清理整治力度，严厉打击保健品诈骗犯罪。严格落实安全生产责任，持之以恒推进“三防三抓三严”，强化安全生产基础工作，提升安全生产和应急处突专业化水平，有效防范和坚决遏制重特大事故。

各位代表，民有所呼，必有所应。我们将继续围绕扶贫解困、生态环保、缓堵保畅、社会建设、民生保障等方面，在新的一年继续抓好总投资267亿元、年度投资133亿元的30件惠民便民实事，确保按期完成，努力让群众生活一年更比一年好！

（八）加强政府自身建设，进一步提高政府治理现代化水平

新时代对政府自身建设提出了更高要求。我们将坚持把加强政治建设放在首位，持之以恒推进学习型、法治型、服务型、务实型、廉洁型政府建设，不断提升政府治理体系和治理能力现代化水平。

新的一年，我们将不断加强政治建设。扎实开展“不忘初心、牢记使命”主题教育，深入学习贯彻习近平新时代中国特色社会主义思想和党的十九大精神，继续在学懂弄通做实上下功夫，树牢“四个意识”，坚定“四个自信”，坚决维护习近平总书记党中央的核心、全党的核心地位，坚决维护党中央权威和集中统一领导，始终在思想上政治上行动上同以习近平同志为核心的党中央保持高度一致。

新的一年，我们将深入推进依法行政。严格遵守宪法和法律，全面贯彻落实法治政府建设实施纲要，把政府活动全面纳入法治轨道。自觉接受人大及其常委会的法律监督和政协的民主监督以及社会舆论监督，广泛听取各民主党派、工商联、无党派人士和人民团体意见。完善集体讨论决定、专家论证、合法性审查、风险评估等机制，不断提高决策法治化民主化科学化水平。深化综合行政执法改革，避免多头执法、多层执法。深入推进政务公开，让权力在阳光下运行。

新的一年，我们将持续提升行政效能。严格按照中省及市委安排部署，确保3月底前全面完成市政府机构改革任务，构建系统完备、科学规范、运行高效的效能体系。大兴学习之风、调查研究之风，不断锤炼“五个过硬”，切实增强“八种本领”。严格落实中央八项规定《实施细则》、省委《实施办法》精神及市委《具体要求》，推动“马上就办、办就办好”再深化、再提速，坚决破除形式主义、官僚主义。运用好“三项机制”，激发调动公务人员担当负责、创新创业的热情。

新的一年，我们将持之以恒反腐倡廉。严格履行全面从严治党责任，完善权力运行制约和监督机制，推进财政预决算公开，紧盯公共资金使用、公共资源交易、公共资产经营、公共工程建设以及扶贫、民生等领域，重拳整治腐败。树立过紧日子思想，严格控制“三公”经费，压缩5%—10%的一般性支出，精打细算，厉行节约，把宝贵的资金用在改善民生上。全体公务人员要始终把纪律和规矩挺在前面，严守廉洁从政各项规定，守住底线、不踩红线、不碰高压线，永葆清廉本色，一心为民服务。

各位代表，新时代是奋斗者的时代，幸福是奋斗出来的。让我们更加紧密地团结在以习近平同志为核心的党中央周围，在省委省政府和市委的坚强领导下，不忘初心、牢记使命，担当实干，奋力谱写追赶超越新篇章，以优异成绩迎接中华人民共和国成立70周年！
